# Cost-effectiveness of a multidisciplinary lifestyle-enhancing treatment for inpatients with severe mental illness: the MULTI study V

**DOI:** 10.1192/j.eurpsy.2022.872

**Published:** 2022-09-01

**Authors:** J. Deenik, C. Van Lieshout, H. Van Driel, G. Frederix, I. Hendriksen, P. Van Harten, D. Tenback

**Affiliations:** 1 GGz Centraal, Scientific Research, Amersfoort, Netherlands; 2 Maastricht University, School For Mental Health And Neuroscience, Maastricht, Netherlands; 3 University Medical Centre Utrecht, Thinc, Julius Center For Health Science And Primary Care, Utrecht, Netherlands; 4 GGz Centraal, Data & Finance, Amersfoort, Netherlands; 5 LivIng Active, -, Santpoort-Zuid, Netherlands; 6 CTP Veldzicht, -, Balkbrug, Netherlands

**Keywords:** physical activity, Lifestyle, schizophrénia, cost-effectiveness

## Abstract

**Introduction:**

Economic evaluations of lifestyle interventions for people with mental illness are needed to inform policy makers and managers about implementing such interventions and corresponding reforms in routine mental healthcare.

**Objectives:**

We aimed to evaluate changes in healthcare costs 18 months after the implementation of a multidisciplinary lifestyle-enhancing treatment for inpatients with severe mental illness (MULTI) versus treatment as usual (TAU).

**Methods:**

In a cohort study (n=114; 65 MULTI, 49 TAU), we retrospectively retrieved cost data in Euros on all patient sessions, ward stay, medication use, and hospital referrals in the quarter year at the start of MULTI (Q1 2014) and after its evaluation (Q3 2015). We used linear regression analyses correcting for baseline values and differences between groups, calculated quality-adjusted life years (QALY) and deterministic incremental cost-effectiveness ratios, and performed probabilistic sensitivity analyses.

**Results:**

Adjusted regression showed reduced total costs per patient per quarter year in favor of MULTI (B=-736.30, 95%CI: -2145.2–672.6). Corresponding probabilistic sensitivity analysis accounting for uncertainty surrounding the parameters showed MULTI was dominant over TAU with a saving in total costs of €417.48 (95%-CI: -2,873.2–2,042.1) against 0.06 improvement in QALY (95%-CI: -0.08–0.20). Costs saving estimates were statistically non-significant showing wide confidence intervals.

**Conclusions:**

Regardless of cost savings, MULTI did not increase healthcare costs while improving QALY and additional previously observed health outcomes. This indicates that starting lifestyle interventions does not need to be hampered by costs. Potential societal and economic value may justify investment to support implementation and maintenance. Further research is needed to study this hypothesis.

**Disclosure:**

No significant relationships.

